# Effect of joint mimicking loading system on zonal organization into tissue-engineered cartilage

**DOI:** 10.1371/journal.pone.0202834

**Published:** 2018-09-12

**Authors:** In-Su Park, Woo Hee Choi, Do Young Park, So Ra Park, Sang-Hyug Park, Byoung-Hyun Min

**Affiliations:** 1 Cell Therapy Center, Ajou University Medical Center, Suwon, Korea; 2 Department of Molecular Science & Technology, Ajou University, Suwon, Korea; 3 Department of Orthopedic Surgery, Ajou University School of Medicine, Suwon, Korea; 4 Department of Physiology, Inha University College of Medicine, Incheon, Korea; 5 Department of Biomedical Engineering, Pukyong National University, Busan, Korea; University of South Carolina, UNITED STATES

## Abstract

Cartilage tissue engineering typically involves the combination of a biodegradable polymeric support material with chondrocytes. The culture environment in which cell–material constructs are created and stored is an important factor. The aim of the present study was to investigate the effects of combined stimuli on cartilage zonal organization which is important to maintain cartilage functions such as lubrication and cushion. For that purpose, we developed a joint mimicking loading system which was composed of compression and shear stress. To mimic the joint loading condition, we manufactured a stimuli system that has a device similar to the shape of a femoral condyle in human knee. The fibrin/hyaluronic acid mixture with chondrocytes were dropped into support made of silicon, and placed under the device. The cartilage explants were stimulated with the joint mimicking loading system for 1 hour per day over the course of 4 weeks. The amounts of GAG and collagen in the stimulated tissue were more than that of the static cultured tissue. Cells and collagen were arranged horizontally paralleled to the surface by stimuli, while it did not happen in the control group. The results of this study suggests that mechanical load exerting in the joint play a crucial role in stimulation of extracellular matrix (ECM) production as well as its functional rearrangement.

## Introduction

Cartilage is an avascular, aneural and alymphatic, but highly specialized type of connective tissue [1, 2]. Both collagen and proteoglycan, which are the primary ingredients of the cartilage, play major roles in the function of cartilage [3]. Proteoglycan contains more than 30 times more water than its molecular weight by holding a strong combination of water, the proteoglycan-water complex is compressed at high pressure by the collagen and thus holds the so-called swollen pressure [4]. Another characteristic of hyaline cartilage is that it is ideally made up of four layers. Such zonal organization of articular cartilage is important for its normal function [5]. The collagen fibers in the superficial layer are arranged parallel to the surface, and thus are designed to withstand the shear stress, and especially by secreting lubricin, it functions as a boundary lubricant. In the deep layer, collagen fibers are arranged vertically to subchondral bone, and thus designed to withstand the compressive force that is put vertically on the cartilage. The cell arrangement and the proteoglycan distribution are also different, and moreover, the way cells in each layer respond to the growth factors and the cytockine turned out to be different as well [6, 7]. It is estimated that these distinguishing biological differences will be important when forming or sustaining a zonal organization of cartilage. structures as similar.

Microstructure of the zonal organization of articular cartilage is believed to be formed by joint loading movement, and is known plays an important role in the performance of functions such as lubrication and cushioning [8]. Zonal organization is assumed to be formed by the mechanical stimuli in cartilage. Such zonal organization can not be found in the cartilage of an infant that does not have the weight bearing on its lower extremites. Once the weight bearing begins, the zonal organization appears, yet shear, compression, and hydrostatic pressure appear inside the joint in various proportions due to weight bearing and muscle tension, and thus the overall action of these kinds of pressures is assumed to cause the zonal organization [9].

Approaches to mimic the zonal structure include methods based on the use of cell therapies, scaffolds, and application of strain fields by using a bioreactor [10]. Cell-based methods typically replicate the native distribution of chondrocyte populations by isolation of zonal chondrocytes. However, in those studies, the material properties of the engineered cartilage were generally not comparable to native hyaline cartilage [11, 12]. Other studies have used multilayered scaffolds to support formation of the cartilage different zonal subpopulations. The multilayered cartilage constructs were produced with zonal chondrocyte organization and qualitatively similar to native hyaline cartilage. However, this effect was not seen with full-depth chondrocytes [13]. Bioreactors for cartilage engineering provide optimized mechanical environments for in vitro functional 3-D tissue development which enhance proliferation and ECM production. [14]. Problems associated with poor diffusion under traditionally static culturing conditions can be mitigated using a mechanical stimulating bioreactor, in which media is forced into the scaffold pores. Although, many bioreactor systems have been investigated for effective biochemical synthesis of cartilage, they have not been investigated for cartilage zonal organization.

Chondrocytes used to engineer cartilage respond to mechanical loading. Physical stimuli are sensed and converted to biochemical signals that regulate fundamental cellular behaviours [15]. It is generally accepted that mechanical loading at physiologically low levels stimulates the biosynthesis of extracellular matrix (ECM) macromolecules [16]. This has motivated the design of various bioreactor systems aiming to provide mechanical stimuli which favour tissue maturation. Mechanical signals generated by bioreactors induce the deformation of the matrix and chondrocytes [15, 16]. Mechanical stimulation of chondrocytes in a specific layer can cause chondrocytes to express specific functions [16]. It is reported that compressive stimulation at the surface induces the production of the surface zone protein Proteoglycan 4 (lubricin) [17]. The use of physiologic dynamic deformational loading or sliding contact loading in chondrocyte based cartilage tissue engineering, results in improved mechanical properties and biochemical content [18–20]. Articular motion is a combination of compressive, tensile and shear deformations; therefore, one can presume that compression alone is unlikely to be a sufficient mechanical signal to generate a hyaline cartilage-like tissue in vitro [18]. It is assumed that simulating of the mechanical stimulus on human joints can create a structure similar to human hyaline articular cartilage. For that reason, we developed a mechanical stimuli system imitating human joint movement, and examined the change of cell arrangement and macromolecular organization on young porcine cartilage explants with zonal free-organization.

## Materials and methods

### Cell isolation and culture

Cylinder shaped cartilage tissues (*d* = 5 mm, *h* = 3 mm) were cut using a biopsy punch (REF 33–35, Miltex, Inc., York, PA) from femoral heads of 2 weeks old pigs within 3 hours of its sacrifice. Pig was obtained from the Garak slaughterhouse (Seoul, Korea). The cartilage explants were washed with phosphate-buffered saline (PBS; Welgene, Daequ, Korea). Explants were digested in 0.2% (w/v) collagenase (Worthington Biochemical, Lake wood, NY) for 5 h at 37°C. Using a cell strainer (70 μm Nylon, Falcon, Franklin Lake, NJ), the cells were filtered, pooled, and centrifuged at 1200 rpm for 10 min. After it was washed twice with PBS, the cell pellet was resuspended in high glucose-Dulbecco’s modified eagles medium (DMEM: Gibco BRL, Grand Island, NY) supplemented with 10% fetal bovine serum (FBS, Gibco BRL), 50 ug/ml ascorbic acid (Sigma-Aldrich, St. Louis, MO), 100 U/mL penicillin G (Gibco BRL) and 100 μg/mL streptomycin (Gibco BRL). The cells were then plated at a density of 1.5 × 10^5^ cells/cm^2^ and placed in a 5% CO^2^ incubator at 37°C. The culture medium was changed every other day. The primary chondrocytes were passaged twice before the experiments were started.

### Preparation of fibrin/HA composite construct

**Fig 1:**
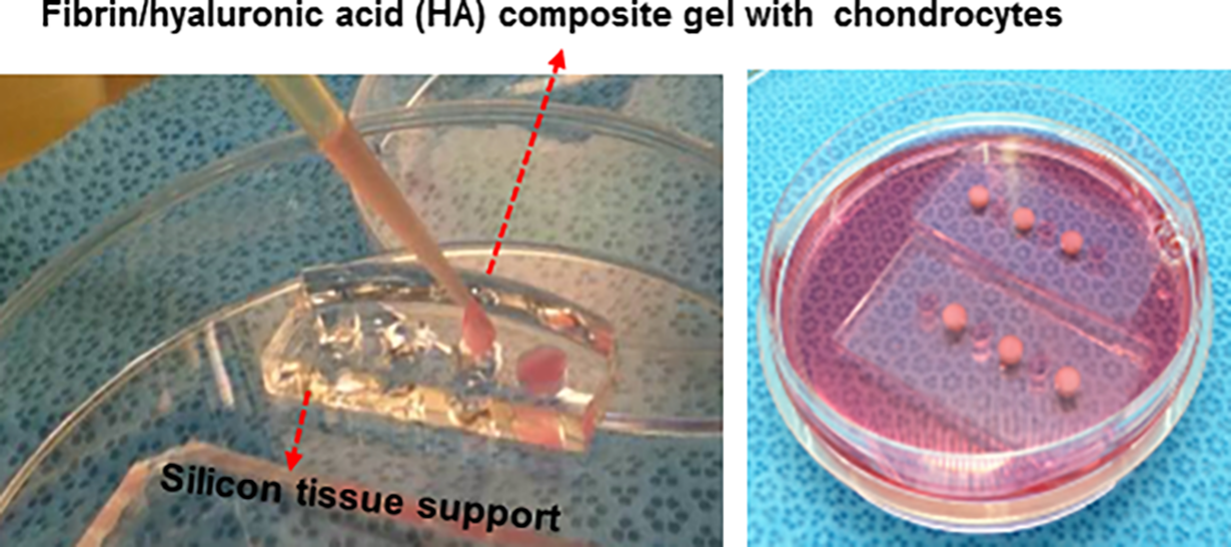
Preparation of fibrin/HA composite gel with cells. The chondrocytes were suspended at 5x10^7^ cells/ml in a solution containing fibrinogen and HA. The fibrin/HA mixture (40 ul) with cells were dropped into support made of silicon (hole size: d = 5mm, h = 3mm).

To prepare the fibrin/hyaluronic acid (HA) composite gel with cells, the chondrocytes were suspended at 5x10^7^ cells/ml in a solution containing 9~18 mg/ml fibrinogen (Green Cross, Korea) and 10 mg/ml HA of 3,000 kDa (LG Chemical Institute, Korea) (Fig 1A). The fibrin/HA mixture (40 ul) with cells was dropped into a support made of silicon (base : curing agent = 20 : 0.7, hole size: d = 5mm, h = 3mm, Sylgard, Sigma-aldrich, MO). The fibrin/HA constructs (size: d = 5mm, h = 3mm) were cultured in high glucose-DMEM supplimented with 100 U/ml penicillin and 100 ug/ml streptomycine, ITS supplement (1.0 mg/ml insulin from bovine pancreas, 0.55 mg/ml human transferrin and 0.5 mg/ml sodium selenite), 50 ug/ml ascorbic acid, 100 nM dexamethasone, 40 ug/ml proline, 1.25 mg/ml bovine serum albumin (BSA) and 100 ug/ml sodium pyruvate.

### Cultivation of cartilage explants

**Fig 2:**
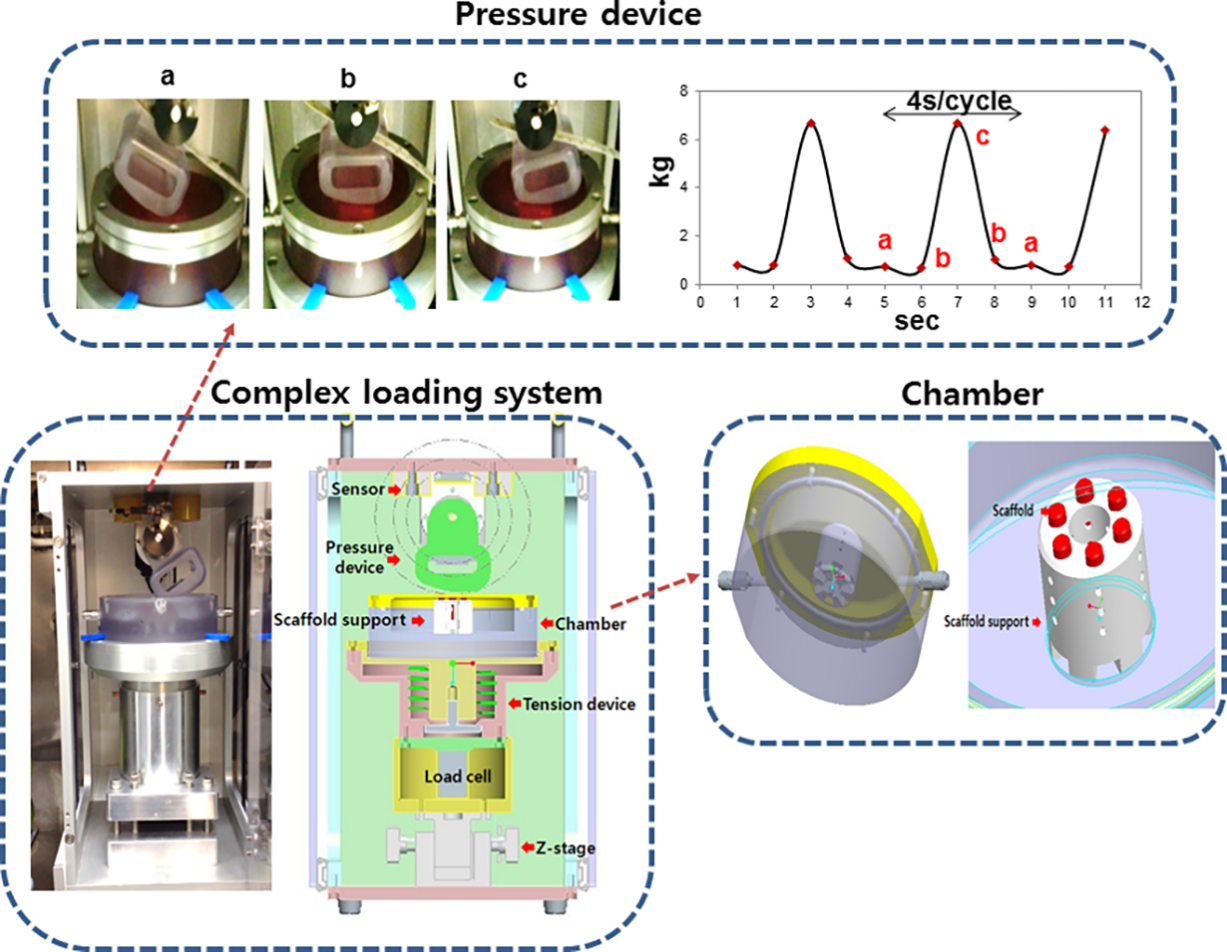
Mimic the joint loading condition. we manufactured the complex loading system that has a device similar to the shape of a femoral condyle in human knee, and the device was designed to produce the shear and compression forces. The chamber for cartilage loading was height-adjustable using Z-stage, and the stimulus was controlled by adjusting the height of the chamber. Cartilage explants were placed under the device and stimulated for 1 hour per day over the course of 4 weeks by the maximum load of 6 kg with shear stress. The motion of device was set-up to be repeated every 4-sec cycle.

The experimental protocol for animal use was reviewed and approved by the Institutional Animal Experiment Committee of Ajou University School of Medicine (AMC-85). The explants were loaded in to a silicon support (base:curing agent = 20:1, Sylgard^®^, Dow corning Co., Midland, MI) (Fig 1). To mimic the joint loading condition, a mechanical stimuli system that has a device similar to the shape of a femoral condyle in a human knee was produced. The device was designed to produce the shear and compression forces (Fig 2). The chamber for cartilage loading was height-adjustable using a Z-stage, and the load was controlled by adjusting the height of the chamber. Cartilage explants were placed under the device and loaded for 1 hour per day over the course of 4 weeks with a maximum load of 6 kg of shear load (0.25 MPa to 0.3 MPa). A control group was cultured without any load in silicon support. A load-cell used to measure the load and calculate the pressure was used to monitor loading, and the motion of device was set-up to be repeated every 4-sec (slow walking speeds).

### Measurement of compressive strength

The pellet cultures were subjected to an unconfined compression test using a Universal Testing Machine (Model H5K-T, H.T.E, Salfords, England). After being placed on the platen of the machine, each sample was compressed at a speed of 1 mm/min. The machine automatically stoped after moving a programmed length between the top and bottom platens. Young’s modulus was calculated and individual compressive strength was collected (n = 4) [21].

### Measurement of chemical components

To measure the GAG contents, the freeze-dried tissues were fully digested for 24 h at 60°C in a papain digestion solution (125 ug/ml of papain, 5 mM L-cystein, 0.1 mM Na_2_HPO_4_, 5 mM EDTA and pH 6.8). Digested samples of 50 ul were loaded in to a 96-well plate, and 200 ul of the DMB solution (8 mg of dimethylmethylene blue (DMB), 2.5 ml of 95% EtOH (Duksan Chemical, Seoul, Korea), 1.5 ml of formic acid, 12.8 ml of 1 M NaOH in 1L of 3DW) was added. After 30 min at room temperature, the absorbance was measured at 530 nm. A 0.1 mg/ml of chondroitin sulfate C was used as a standard. Total collagen contents of tissues were measured by a chloramine-T hydroxyproline assay [22, 23]. All reagents used for the biochemical assay were purchased from Sigma Chemical Co.

### Histological analysis

After 4 weeks of loading, the tissues were fixed with 4% formalin and embedded in paraffin wax and sectioned at a 4 um thickness. The sections were deparaffined with xylene, hydrated in decreasing concentration of EtOH in 3DW (3–5min at concentrations of 100, 95, 85, 80, 75 and 0%), and stained with Hematoxylin/Eosin (H&E), Safranin-O, Sirius Red and Lubricin (IHC) to identify cell arrangement, macromolecular organization and cartilage surface proteins. After staining, the slides were mounted with a mounting solution before microscopic observation (Nikon E600, Japan). The sections were reacted with a DAB solution (Golden Bridge International) and counterstained with Mayer’s hematoxylin (YD Diagnostics, Seoul, Korea).

### Scanning electron microscope (SEM) for collagen-linking fiber

To confirm the macrofibrillar collagen network, the samples were incubated for 5 hours in 2 M CaCl_2_ solution at 37°C for extraction of proteoglycans and then fixed in 4% glutaraldehyde for 12 hours. Fixed samples were washed with PBS and dehydrated in increasing concentrations of acetone (Duksan Chemical) in 3DW, and critical point drying was performed. After drying, the tissues were freeze-fractured using liquid nitrogen (LN_2_), and then cut vertically into the cartilage surface. The sections were coated with gold using a sputter coater (Sanyo Denshi, Tokyo, Japan) and examined with a Scanning Electron Microscope (SEM; JEOL Ltd. JEOL, Japan)

### Statistical analysis

Statistical analyse performed by a one-way variance (ANOVA) test with the post hoc least significant difference (LSD) multiple comparison test using SPSS 12.0.1 (SPSS, Chicago, IL). The values of result were presented as means with standard error of the means (SEM), and *P* values < 0.05 were regarded as statistically significant.

## Results

### Gross observation and mechanical strength analysis

**Fig 3:**
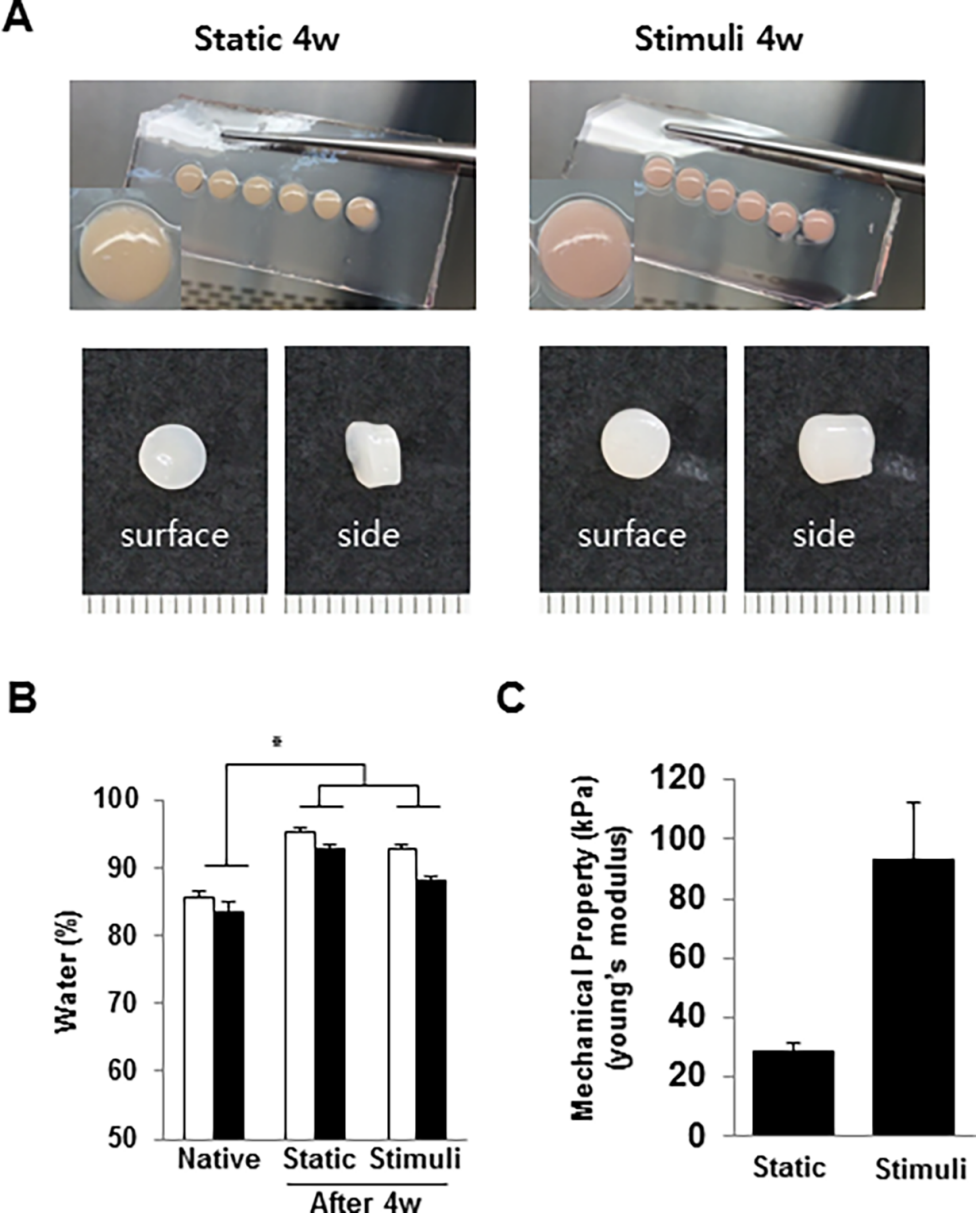
Gross observation and mechanical strength analysis. (A) The tissues were gross observed after 4 weeks of stimuli. (B) These tissues were divided into upper and lower by swollen portion, and the wet weight and dry weight were measured before and after freezing-dry, respectively. In the cultivated tissues, the water contents were increased compared with native cartilage, and the static cultured tissue has the largest amount of water (* *p* < 0.05). (C) Young’s modulus measurement of tissues at 4 weeks (n = 3).

After 4 weeks of cultivation, the surface and bottom of tissue were swollen in the static cultured group. Whereas, in the loaded group, the surface of tissue maintained the same flatted surface as was observed before they were cultured, and the bottom of tissue was deformed by the loading (Fig 1). These tissues were divided into upper and lower parts, and the wet weight and dry weight were measured before and after freezing-dry, respectively. In the cultured tissues, the water contents were increased compared with native cartilage, and the static cultured tissue had the largest amount of water (Fig 3A and 3B). There was more water in the upper layer compared to the lower layer, and a significant difference was observed between the upper and lower layer in the native and loaded groups. After loading for 4weeks, the loaded group was observed to be more opaque than the static (control) group. We expected that more ECM production in the loaded group than the control group. This study estimated the Young modulus of the stimulated tissues to be 93.15 kPa.

The mechanical properties of the stimulated tissues were higher than that of the static group (28.49 kPa) by three-fold (Fig 3C). The young’s modulus of the native pig cartilage is 2.6 MPa.

### Biochemical contents

**Fig 4:**
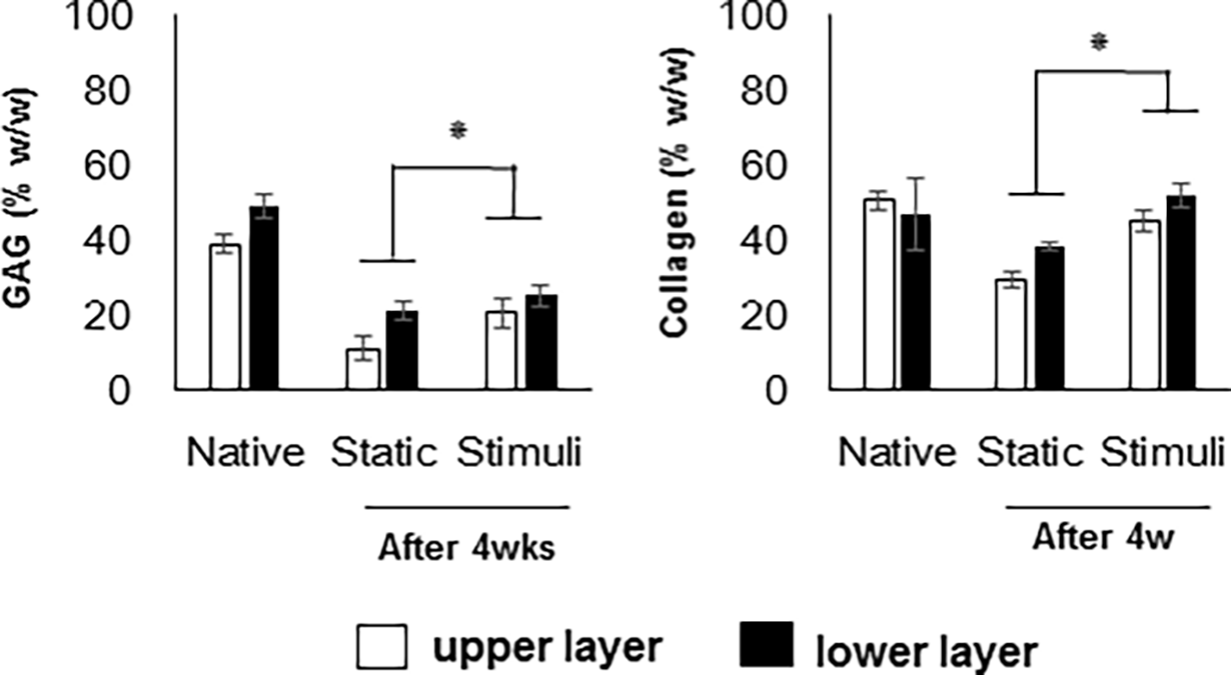
Measurement of the amount of GAGs and collagen in the constructs. The total amount of GAGs and collagen was normalized by dry weight (mg) of each specimen. Data were presented as mean values with SD from three independent experiments (n = 3). Statistical significance was analyzed between different culture time groups (*p < 0.05).

The GAG and collagen contents of the each piece were measured with a dimethylmethylene blue (DMB) and chloramine-T hydroxyproline assay, respectively. The mesured values were normalized to dry weight. In both groups, the lower layer has more biochemical contents than the upper layer, and a significant difference was observed between the upper and lower layers. In the loaded group, more GAG and collagen were present than control group, but all results of both groups were lower in comparison with the results of native cartilage (Fig 4). The transition from the superficial to the calcified zone is characterised by an increase in GAG content and compressive strength.

### Histological change of cartilage explants

**Fig 5:**
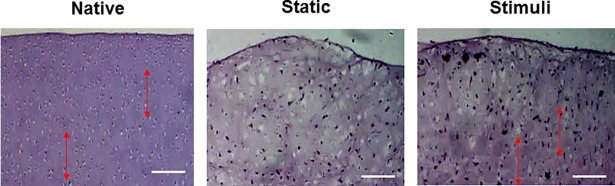
Cell arrangement was confirmed by H & E staining. After cultivation for 4weeks, the cells were randomly arranged in the surface of static cultured tissue, while cells were vertically arrangement (arrow) in stimuli tissue. Scale bar is 100 μm.

To demonstrate the change of cell arrangement caused by loading, tissues were stained with H&E. In native cartilage, the cells were randomly arranged. (Fig 5) After cultivation for 4 weeks, the cells were randomly arranged in the static cultured tissue, while cells were vertically arranged in the loaded tissue. In the control group, cell-free lacuna were found more often than in the loaded group.

**Fig 6:**
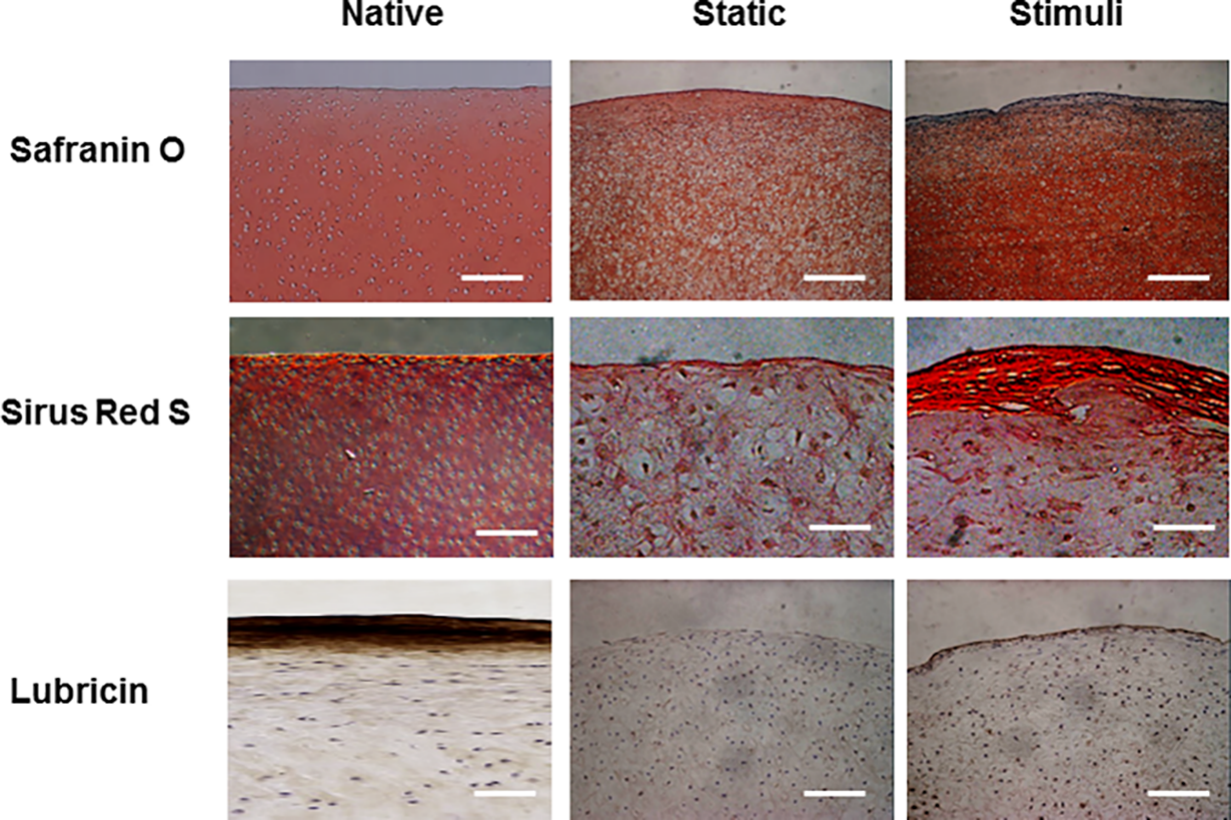
The change of cell arrangement, GAG synthesis, cartilage surface marker expression and collagen fiber arrangement by stimulation. GAG expression was confirmed by Safranin-O staining. Stimulated tissues were express more amount of GAG. Scale bar is 400 μm. Lubricin (superficial zone protein) expression was confirmed by immunohistochemistry. Scale bar is 400 μm. In the stimuli group, lubricin expression was intensely expressed in the surface of tissue. Total collagen fiber arrangement was confirmed by Sirius Red staining. In the stimuli group, total collagen fiber was expressed intensely and make horizontal with the surface. But, static group had shown that collagen was weakly. Scale bar is 100 μm.

The sGAG expressions were confirmed through the Safranin-O staining, and expressed more amount of sGAG in the stimulated tissue (Fig 6). In the surface of tissue, however, GAG showed no differences between the static and stimuli groups. In the bottom of tissue, more and larger sized lacuna that means cartilage maturation and cell clustering that observed in the deep zone of organized cartilage was found in the stimuli group. Also, GAG expression was more in the stimuli group.

### Collagen alignment and collagen fiber-linking network by loading system

**Fig 7:**
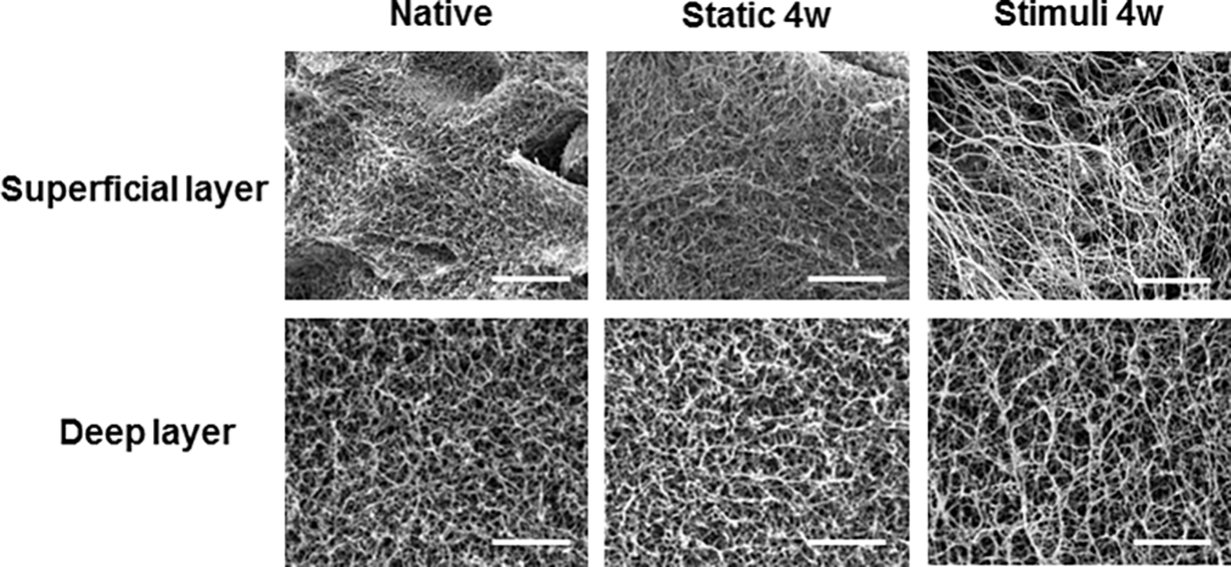
The formation of collagen fiber arrangement by stimulation. The collagen fibers was confirmed by scanning electron micrographs analysis. After 4 weeks of cultivation, the fibers were not formation at static group. In the combined group, the images had shown that collagen fibers were thickly formed on the surface. Scale bar is 5 μm.

To confirm the change of collagen alignment by loading, the tissue sections were stained with Sirius Red S. The collagen was generally observed to be vertically oriented in the surface of the loaded tissue, while it was generally randomly oriented in the surface of the static cultured tissue (Fig 6). The direction of collagen alignment was consistent with the cell arrangement and orientation. In the bottom region of loaded, the collagen of stimulated tissue was more intensely expressed in comparison with static cultured tissue. Collagen linking-fibers were confirmed using SEM analysis after removing the GAG in cartilage tissue. In the native tissue, the collagen fibers in the superficial layer were more closely packed and oriented parallel to the cartilage surface, while the fibers on the bottom zone were more loosly packed than those in the superficial layer and showed a more isotropic arrangement. (Fig 7) After 4 weeks of static culture, the collagen fiber-linking network was loosely packed in both the superfishal layer and the deep layer, and the parallel arrangement in the superficial layer disappeared in comparison with the superficial layer of native cartilage. In the loaded tissue, however, the results had shown that more collagen fibers were loosly and sparsely distributed in the superficial and deep layers. Especially, the vertically oriented collagen fibers gradually appeared in the deep layer.

## Discussion

Forces transmitted across the knee joint during normal walking range between 2 and 3 times body weight [24]. Since the shape of a human knee is not round with single axis of rotation but is an ellipsoid with many axes of rotation, when the knee moves through its flexion and extension, various amounts of shear, compression, and hydrostatic pressure are generated. However, the exact magnitude and composition of the pressure is not well established. Also, the exact pressure and intensity of the load needed to induce zonal organization is unkown. In addition, the direction that loading has to be applied to in order to construct the zonal organization of the normal cartilage is also unknown. This study attempted to simulate the shape of the a human knee, and although dimension vary, it is assumed that the pressure pattern of the human knee was recreated.

In this study, cartilage explants (*d* = 5 mm, *h* = 3 mm) were loaded to 6 kg with shear stress (repeated every 4-sec per cycle). To induce of zonal organization on articular cartilage tissue, mechanical motion needs to more accurately mimic the joint movement [12]. In this study, we manufactured a loading system that can apply complex loading mimicking human joint loading, and we confirmed the change of cell arrangement and macromolecular expression due to loading on porcine cartilage explants. Compression and shear stress are typical stimulii that are during joint loading. Many studies reported that compression enhances the ECM synthesis on chondrocytes and cartilage tissue or chondrogenic differentiation of MSCs [25]. A combination of shear and compression is more effective in maintaining or inducing chondrogenic potential than each individually application. In this study, immature cartilage developed into mature cartilage by changing protein secretion and collagen fiber arrangement in response to mechanical stimulation. According to previous studies, immature cartilage integrates better with the surrounding cartilage, and is able to mature more easily [2, 7]. The results of our study are encouraging for the treatment of cartilage defects. It is important for tissue engineered cartilage implants not only to provide for construct survival, but also to adequately respond to loading after implantation. The Young’s modulus of the loaded group (93 kPa) was 3.3 times higher than that of the static group (28 kPa). It was demonstrated that in a loaded culture environment tissues became biochemically and mechanically saturated (Fig 3C). The GAG and collagen contents, as well as mechanical strength were considered reliable indices of the usefulness of this construct for clinical treatment. According to the result of this study, the upper layer of the loaded group that was cultured for 28days had 54% of native GAG content, and 90% of native collagen content (Fig 4). In comparison with the static cultured group, the application of mechanical stimulation resulted in increased GAG content of cartilage explants (approx. 200% of the static controls, *P <*0.05) and the collagen content (approx. 150% of the static controls, *P <* 0.05) (Fig 4). Furthermore, a suitable distribution of mechanical stress and synovial factors influenced the remodeling of the articular cartilage. These characteristics may make it possible to rapidly and suitably regenerate cartilage bone structure *in vivo*. Recently, several studies demonstrated that lubricin was induced by mechanical stimulation in the superficial zone of cartilage. The lubricin was expressed on the surface of explanted cartilage disks in a continuous compressure system [26–28]. One of the major limitations of the these works is that the bioreactor cultivated cartilage lacked other important features of articular cartilage, including the characteristics of a middle and deep zone architecture. Our study shows that, the top region of explants was directly stimulated through loading by the femoral condyle shaped device (Fig 2), and showed the biggest change. In the upper layer of tissue, cells were arranged horizontally paralleled to the surface (Fig 5) and ECM components (GAG, collagen) increased due to loading (Fig 6). These studies also suggest that joint mimicking loads could induce lubricin expression on the surface of cartilage tissue (Fig 6). Therefore, we considered this loading system could also be used to influence lubricin expression. The lubricin expression might be induced by initiation of relative motion between the articular surfaces, and has multiple functions in articulating joints that include the protection of surfaces and the control of synovial cell growth [29]. Particularly, thickly collagen fibers were observed on upper layer of stimulated group (Fig 7). These characteristics may make it possible to rapidly and suitably regenerate cartilage bone structure in vivo’.

## Conclusion

Joint mimicking loads produced the larger amounts of GAG and collagen, and aligned the cells horizontally paralleled to the surface, while it did not happen in the static cultured tissue. The results of this study suggests that mechanical load exerting in the joint play a crucial role in stimulation of ECM production as well as its functional rearrangement.
